# Higher Pre‐ and Post‐Monsoon Temperatures and Their Impact on Child Undernutrition in Bangladesh

**DOI:** 10.1111/mcn.70176

**Published:** 2026-03-16

**Authors:** Syed Shahadat Hossain, Souvik Ghoshal Aranya, Jahida Gulshan, Md. Bazlur Rashid

**Affiliations:** ^1^ Institute of Statistical Research and Training (ISRT) University of Dhaka Dhaka Bangladesh; ^2^ Bangladesh Meteorological Department Dhaka Bangladesh

## Abstract

Child malnutrition remains a critical public health concern in Bangladesh, with emerging evidence linking climatic variability to nutritional outcomes. This study examines the impact of unusual seasonal temperature deviations, particularly in the pre‐ and post‐monsoon periods, on child malnutrition. District‐level time series climatic data from 2007 to 2018 were analyzed alongside the pooled cross‐sectional children's data, the Multiple Indicator Cluster Survey (MICS) of 2012 and 2019, to assess these effects. Factor analysis and multilevel logistic regression model were used for analyzing the data where factor analysis identified the pre‐ and post‐monsoon seasons as a dominant weather factor, and multilevel logistic regression evaluated its association with malnutrition after adjustment for other individual‐level, household‐level, and maternal characteristics. Results indicate that higher‐than‐usual temperatures in pre‐ and post‐monsoon seasons significantly increase stunting, while lower‐than‐usual monsoon temperatures do the same to both stunting and wasting. These findings suggest that deviations from historical climatic norms may negatively affect child nutrition. Strengthening climate‐sensitive nutrition policies and early warning systems is essential in mitigating the impact of unseasonal temperature anomalies on child health in Bangladesh.

## Summary

Higher pre‐ and post‐monsoon temperatures significantly increase child stunting with important cumulative and lagged effects, while monsoon temperatures are negatively associated with both stunting and wasting underscoring the need for climate‐resilient, district‐targeted nutrition strategies.

## 1 Introduction

In an era characterized by mounting global environmental apprehensions, the complex connection between climate change and human well‐being has risen to the forefront of both research and policy considerations. Climate change is an ongoing and significant global issue characterized by long‐term shifts in weather patterns, including temperature, precipitation, and extreme weather events. Statistical analysis of historical weather data is a fundamental step in understanding the patterns of changes and their associations in Bangladesh. Advancements in climate modeling techniques have enabled researchers to forecast weather variability in Bangladesh more accurately. Studies by Blanc and Schlenker (2017), Islam et al. (2022), and Kamruzzaman et al. (2023) used climate models to predict future temperature and rainfall patterns, providing valuable insights for policymakers and stakeholders in planning adaptation and mitigation measures.

Recent advancements in climate modeling have improved the precision of weather forecasts, enabling researchers to better analyze temperature and rainfall trends and inform adaptive strategies (Blanc and Schlenker 2017; Islam et al. 2022; Kamruzzaman et al. 2023). Several studies have documented increasing variability in these weather patterns (Khan et al. 2019; Alam et al. 2023; Haider et al. 2024), with projections by Rashid et al. (2019, 2020, 2021) highlighting a sustained rise in maximum temperatures during the pre‐ and post‐monsoon seasons.

The climatic changes observed in Bangladesh manifest in diverse and geographically nuanced forms—prolonged heatwaves, irregular rainfall, intra‐seasonal droughts, and winter precipitation (Tuihedur Rahman et al. 2018). These shifts have serious implications: prolonged heat can strain ecosystems and human physiology (Dastagir 2015; Rahman et al. 2023), while unpredictable rainfall exacerbates flooding and soil erosion, undermining agriculture and livelihoods (Chowdhury and Khan 2015; Sarker et al. 2012). Cumulative impacts of extreme weather across successive years compound vulnerabilities in already at‐risk regions (Moon 2023; Godde et al. 2021), further complicated by ecological disruptions such as habitat loss and species migration (Rahman and Alam 2020).

Beyond the environmental and agricultural sectors, climate variability has direct and indirect health consequences. Past research has linked weather fluctuations with the spread of vector‐borne diseases (Hundecha and Merz 2012; Hossain and Majumder 2018) and plant diseases critical to food security (Kamei and Singh 2021). However, its influence on nutrition, especially among children, remains a pressing and underexplored issue. Malnutrition continues to affect a significant portion of children in Bangladesh, with stunting (28%), underweight (22.6%), and wasting (9.8%) rates reported by MICS 2019. These disparities are compounded by climatic inequalities across regions (Acharya et al. 2020; Ghosh et al. 2021).

Recent evidence points to the adverse effects of extreme temperatures on child nutrition. Raza et al. (2025) highlighted that rising heat threatens decades of progress in reducing stunting. Yet, most existing studies address climate impacts using isolated or short‐term variables, failing to capture the cumulative effects of unstable seasonal patterns over time. Malnutrition, by nature, unfolds over extended periods, shaped by climate factors with time‐lagged effects—a dimension largely overlooked in current research. To address this research gap, the present study links district‐level, high‐resolution ENACTS‐BMD (0.05°) weather data with MICS child anthropometry, incorporating explicit 1‐ and 2‐year seasonal lags. It further applies factor analysis (FA) to mitigate multicollinearity among correlated weather variables before multilevel modeling. This approach enables the identification of pre‐ and post‐monsoon temperature as key climatic influences on stunting and wasting, a relationship that, to our knowledge, has not been examined previously in this context.

So the objectives of this study can be delineated as: (i) To assess the influence of climatic variability—particularly pre‐ and post‐monsoon temperature—on child nutrition outcomes, focusing on stunting and wasting in Bangladesh. (ii) To capture the cumulative and time‐lagged effects of weather patterns by linking high‐resolution ENACTS‐BMD data with MICS child anthropometry at the district level. (iii) To apply factor analysis and multilevel modeling for identifying key climatic factors while addressing multicollinearity among correlated weather variables. (iv) To contribute to the empirical understanding of how long‐term climatic fluctuations interact with child nutrition outcomes, providing a methodological basis for future research in similar settings.

To this end, a district‐level dataset linking nutritional outcomes with localized weather indicators was applied to characterize seasonal temperature and rainfall trends from preceding years of recorded undernutrition, enabling a comprehensive analysis of the role of prolonged seasonal instability. This research contributes to the growing discourse on climate‐informed nutritional policy, offering actionable insights to mitigate the health impacts of climate change in Bangladesh and the findings aim to inform both national and international strategies for mitigating the nutritional impacts of climate change.

## 2 Methodology

### 2.1 Data and Variables

This study uses secondary data from two rounds of the Bangladesh Multiple Indicator Cluster Survey (MICS) conducted in 2012–13 and 2019, the global flagship household survey program of UNICEF. Detailed sampling design, data collection, and measurement procedures are available in the official Bangladesh MICS reports (Bangladesh Bureau of Statistics BBS and UNICEF Bangladesh 2014; Bangladesh Bureau of Statistics BBS and UNICEF Bangladesh 2019). District‐level climatic data for the period 2007–2018 were obtained from the Bangladesh Meteorological Department (BMD).

The analysis was restricted to children aged 24–59 months with complete anthropometric, socio‐demographic, and district‐level climate data, resulting in a final analytical sample of approximately 9000 children. Exclusions were due to age < 24 months, missing or implausible anthropometric values, missing key covariates, and inability to merge with climate data. Survey weights were applied, and excluded and included children did not differ substantially in key demographic characteristics.

The primary outcome variables were stunting (low height‐for‐age) and wasting (low weight‐for‐height) among children under 5 years of age. Anthropometric measurements were collected using standardized procedures and converted into Z‐scores according to the World Health Organization (WHO) Child Growth Standards. Children with height‐for‐age or weight‐for‐height Z‐scores below −2 standard deviations from the WHO reference median were classified as stunted or wasted, respectively.

The main exposure variables were climate indicators, including temperature and rainfall. Localized daily weather data from 2007 to 2018 were sourced from the Bangladesh Meteorological Department (BMD) using the high‐resolution (0.05° × 0.05°) gridded dataset from the Enhancing National Climate Services for Bangladesh Meteorological Department (ENACTS‐BMD; Acharya et al. 2020). District‐specific weather variables, including a highest daily temperature and daily rainfall, were aggregated to derive average seasonal temperature and aggregate seasonal rainfall for each year. Four key seasons were defined based on regional climatic patterns: pre‐monsoon (March–May), Monsoon (June–August), Post‐monsoon (September–November), and Winter (December–February).

To align climatic exposure with the MICS survey years, seasonal weather variables were assigned lag identifiers. For example, the average highest temperature during the Monsoon season in 2011 was treated as the 1‐year lag Monsoon temperature exposure for children surveyed in the 2012 MICS. Similarly, aggregate pre‐monsoon rainfall in 2015 was considered the 2‐year lag pre‐monsoon rainfall exposure for children surveyed in the 2019 MICS. Consistent with the study's analytical framework, the analysis focused on climate exposures with a 2‐year lag. Accordingly, children younger than 24 months at the time of survey were excluded, as they did not have complete exposure to the two preceding years of climatic conditions. These inclusion and exclusion steps are summarized in the data flow diagram.

Socio‐economic and demographic covariates included access to safe drinking water (Yes/No), access to improved sanitation (safe toilet: Yes/No), access to handwashing facilities (Yes/No), household wealth quintile (Poorest, Poorer, Middle, Richer, Richest), child's age in months (24–35, 36–47, 48–59), mother's education (No education, Primary, Secondary, Higher), mother's age at first marriage (continuous), and mother's current age (15–19, 20–24, 25–29, 30–34, 35–39, 40–44, 45–49).

All individual‐level MICS variables were merged with district‐level seasonal climate indicators to create the final analytical dataset, in which children were linked to district‐specific lagged climate exposures. This integrated dataset captures both individual‐level nutritional outcomes and socio‐demographic characteristics alongside spatially and temporally varying district‐level weather conditions.

### 2.2 Statistical Methodology

The paper employs factor analysis to characterize correlated weather variables and seasonal patterns, and multilevel mixed‐effects models to assess their association with child nutrition across demographic groups. Descriptive and exploratory analyses were used to examine nutrition trends, evolving climatic conditions, and their relationships with nutritional outcomes. All analyses were carried out considering the sampling weights provided with the MICS data.

In this paper, factor analysis (FA) (Hox and Bechger 1998; Kaiser 1960) is used to uncover latent factors that represent shared variability among groups of correlated weather variables, allowing us to isolate dominant seasonal influences. With a total of 16 variables, FA allows us to extract a set of independent factors that encapsulate dominant seasonal weather characteristics. This approach helps determine whether specific factors, particularly those dominated by pre‐monsoon and post‐monsoon temperatures, emerge as isolated contributors to climate variability.

We employed a multi‐variable approach of multi‐level logistic regression model (Wong and Mason 1985). In this process, we considered the household‐level data to be nested within district and then within the time points (MICS rounds). This will account for the variation due to the apparent factors districts and time, while the other explanatory variables will be treated as fixed effect in the model. Instead of considering the seasonal rainfall and temperature variables at different lags as explanatory variables in the model, we used the weather factors identified by the FA. This was advantageous as it reduced the number of weather‐related variables, mitigated the multicollinearity challenge (Lafi and Kaneene 1992) by making the weather variables independent and characterized weather patterns in an interpretable way.

The multi‐level logistic regression model (Equation 1) analyzed in the study is

(1) $$\ln\;\left(\frac{{\hat{P}}_{\mathrm{ijt}}}{1-{\hat{P}}_{\mathrm{ijt}}}\right)=\beta_{0\mathrm{jt}}+\beta_{1}X_{\mathrm{ijt}}+\beta_{2}F_{\mathrm{ijt}},$$

where, $Y_{\mathrm{ijt}}$ denotes the binary outcome of stunting status of the $i^{\text{th}}$ child within district j at time t, taking value 1 (stunted) for children whose height‐for‐age (HAZ) z‐score falls 2 standard deviations (SD) below the median of the reference population, and ‘zero’ otherwise; ${\hat{P}}_{\mathrm{ij}}$ represents the predicted probability of the binary outcome $Y_{\mathrm{ijt}}\;$being 1, $X_{\mathrm{ijt}}\;$denotes the explanatory variables; $F_{\mathrm{ijt}}\;$denotes the weather factors identified in the FA; and $\beta_{1}$ and $\beta_{2}$ are the coefficients estimated by the logistic regression model, while $\beta_{0\mathrm{jt}}$ is the random intercept defined as $\beta_{0\mathrm{jt}}=\;\beta_{0}+u_{j}+v_{t}$ with random components $u_{j}$ and $v_{t}$ incorporating district variability and MICS rounds variability respectively.

### 2.3 Ethics Statement

This study uses secondary data on children from a public database, where all personal information has been anonymized; thus, ethical approval is not required.

## 3 Results

In this study, a comprehensive exploration of several climatic factors has been undertaken, encompassing mean seasonal temperatures and aggregated seasonal rainfall across 64 administrative districts of the country over the period of 2007 to 2018. This thorough analysis has been intended to establish an inclusive understanding if there exists any pattern in the change and variation in the weather variables as well as in a few of the weather events across time and districts in Bangladesh. As already mentioned, each of these climatic variables has been rigorously generated for each district across multiple years leading up to the nutrition survey.

### 3.1 Spatio‐Temporal Distribution of Weather Variables in Bangladesh

The district and season‐wise mean temperature and aggregate rainfall of the country during 2007–2018 were analyzed for descriptive features (Annex A1 presents a holistic visualization).

#### 3.1.1 Temperature

It is observed (please see Figure A1) that the district‐wise climatic profile of Bangladesh between 2007 and 2018 demonstrates significant trends in temperature, extreme weather events, and rainfall patterns. In pre‐monsoon months, temperatures typically range between 25°C and 30°C, with exceptions in the eastern districts of Sylhet, Sunamganj and Rangamati exhibiting relatively lower temperatures. During the monsoon season, temperatures show a subtle upward trend with all the districts show similar patterns, post‐monsoon temperatures experience a slight upward trend overall, with exceptional years like 2008 and 2011 recording lower temperatures. Winter temperatures vary spatially, with districts like Cox's Bazar consistently reporting higher mean temperatures, while other northern districts exhibit lower averages.

#### 3.1.2 Rainfall

The pre‐monsoon rainfall has wide‐ranging variability across districts, with some eastern districts consistently receiving higher totals (Figure A1). A pattern across different districts and seasons underscores the spatial and temporal variations in precipitation. These variations include instances of heightened winter rainfall in recent years in some regions. Such changes can have implications for local water resources and ecosystems. Monsoon rainfall also varies significantly, with Eastern districts like Sunamganj, Sylhet, Cox's Bazar, and Chattogram consistently receiving elevated levels of rainfall. Post‐monsoon rainfall generally decreases, with exceptions in a few districts during the specific year 2017.

### 3.2 Weather Variables of Two Preceding Years of MICS Rounds

For each survey round, we considered four seasons—pre‐monsoon, Monsoon, Post‐monsoon, and Winter—and constructed 16 variables representing seasonal rainfall and temperature with a lag of one and 2 years. This section examines the interdependence among these seasonal weather variables to understand their relationships and potential redundancies. By analyzing correlations and dependencies among these variables, we aim to identify whether specific seasonal factors, particularly pre‐monsoon and Post‐monsoon temperatures, stand out as dominant and isolated contributors to climate variability. This assessment informs the selection of key weather variables for modeling their impact on child nutrition outcomes.

### 3.3 Inter‐Dependence Among Seasonal Weather Variables: Correlation

Interdependence among seasonal weather variables was studied to understand their interconnectedness and potential implications for further analysis, particularly in regression models. Bivariate correlation analysis was conducted (a correlation plot is given in Annex Figure A2, the detail results are given in Annex Tables A1 through Table A3).

There are significant correlations between rainfall variables at 1‐ and 2‐year lags. Rainfall of the same season across years displays moderate to strong positive correlations, indicating consistent seasonal rainfall patterns, while correlations across different seasons suggest continuity in precipitation behavior (Figure A2). Similarly, temperature variables within the same season at different lags exhibit moderate to strong positive correlations, reflecting stability in seasonal temperature trends. Moderate correlations across seasons—particularly between pre‐ and post‐monsoon temperatures—indicate possible carryover effects between seasons. A negative correlation between winter and pre‐monsoon temperatures at a 2‐year lag suggests that extreme highs and lows may occur within the same year, signaling dual‐direction weather extremes. The bottom‐left panel also shows consistent but low negative correlations between rainfall and temperature variables at 1‐ and 2‐year lags.

**Figure 1:**
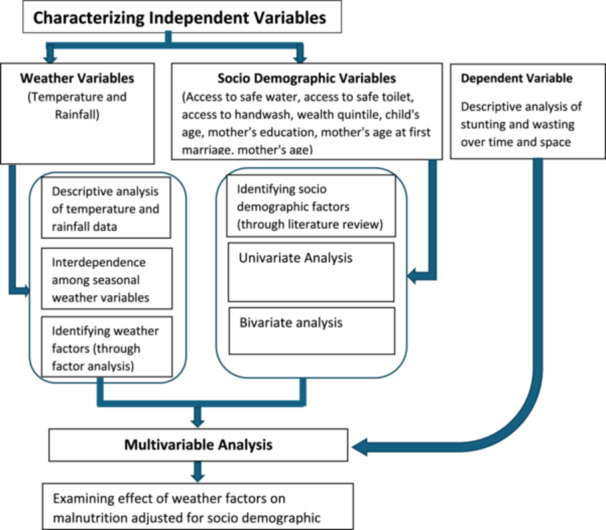
Flow chart of the study.

Overall, these patterns highlight the temporal and seasonal interconnections of rainfall and temperature. Though not uniformly strong, the observed associations emphasize the need to consider these variables jointly rather than separately, as using individual weather variables in regression analyses may yield misleading results due to multicollinearity among them. Figure 1 shows the flow chart of the entire study that summarizes the different types of independent and devependent variables and the analyses performed using those variables.

#### 3.3.1 Factor Analysis of Weather Variables

**Figure 2:**
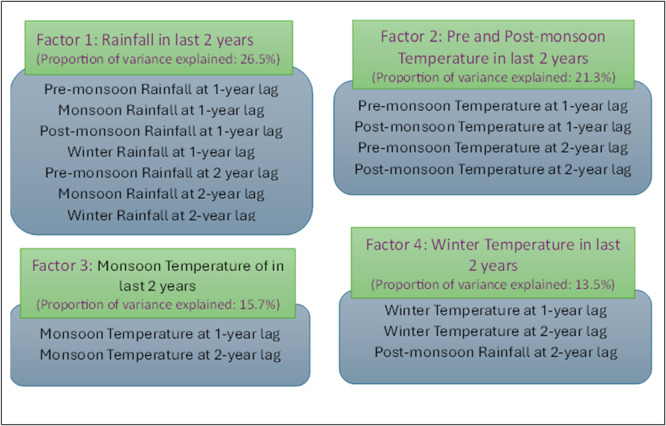
The first four factors of the FA.

Given the strong correlations among the lagged rainfall and temperature variables across different seasons, Figure 2 outlines four principal factors along with their associated weather variables obtained through factor analysis (Hox and Bechger 1998). To enhance interpretability, each factor has been assigned a descriptive name.

Notably, the first factor is predominantly composed of all rainfall variables, except for post‐monsoon rainfall at a 2‐year lag. The second factor distinctly groups pre‐ and post‐monsoon temperatures from both 1‐year and 2‐year lags, effectively isolating these four variables. The third factor similarly isolates monsoon temperatures from both time lags. Meanwhile, the fourth factor is composed of winter temperatures at the two time points, along with post‐monsoon rainfall at a 2‐year lag. This structured grouping of variables provides meaningful insights into the seasonal weather patterns influencing climate variability and their potential links to nutrition outcomes.

### 3.4 Effect of Weather Variables on Stunting and Wasting

**Figure 3:**
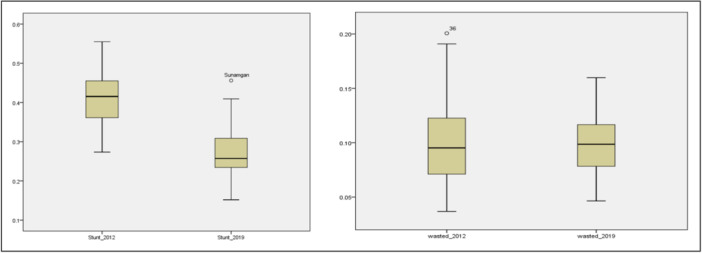
Box plots showing variability of stunting and wasting among districts.

Using district‐level MICS data from 2012 to 2019, we analyze trends in stunting and wasting. Figure 3 shows that stunting was alarmingly high in 2012, with half of the districts exceeding 42% and some surpassing 55%. By 2019, stunting declined notably, with most districts falling below the 2012 median, except for outliers such as Sunamganj. In contrast, wasting showed little improvement, with median prevalence nearly unchanged (9.5% in 2012 vs. 9.8% in 2019). Changes in quartiles and maximum values suggest some distributional shifts, indicating that while stunting has improved nationally, wasting remains largely persistent, with regional disparities still evident.

### 3.5 Association of District Level Under‐Nutrition Prevalence With Weather Variables

District‐level differences in malnutrition suggest factors beyond direct interventions. To examine links with weather, we correlated stunting and wasting prevalence with district‐level weather variables at various time lags (Table 1). Stunting in 2019 was associated with pre‐monsoon temperatures (1‐ and 2‐year lags) and winter rainfall (2‐year lag), while both 2012 and 2019 stunting correlated with pre‐monsoon rainfall at 2‐ and 4‐year lags, highlighting the influence of past weather patterns. Wasting, in contrast, showed no significant links with temperature but was associated with pre‐monsoon and winter rainfall at 1‐ and 2‐year lags, suggesting rainfall impacts wasting more than temperature.

**Table 1:** Bivariate correlation between seasonal weather variables with stunting and wasting prevalence.

		Stunting		Wasting	
		2012	2019	2012	2019
Pre‐monsoon temperature	At 1 year lag	−0.13	−0.26*	−0.01	0.00
	At 2 years lag	−0.15	−0.27*	−0.16	−0.05
Monsoon temperature	At 1 year lag	0.12	−0.07	0.17	0.02
	At 2 years lag	0.01	−0.08	0.03	0.03
Post‐monsoon temperature	At 1 year lag	−0.01	−0.08	−0.01	−0.08
	At 2 years lag	0.01	0.03	0.01	0.03
Winter temperature	At 1 year lag	−0.04	0.02	0.08	−0.06
	At 2 years lag	−0.02	0.05	0.12	−0.07
Pre‐monsoon rainfall	At 1 year lag	0.10	0.20	0.25*	−0.16
	At 2 years lag	0.21*	0.28*	0.25*	0.01
Monsoon rainfall	At 1 year lag	−0.00	0.14	0.11	0.07
	At 2 years lag	0.16	0.15	0.18	−0.02
Post‐monsoon rainfall	At 1 year lag	−0.03	0.03	−0.03	0.09
	At 2 years lag	0.02	−0.10	0.03	−0.17
Winter rainfall	At 1 year lag	0.11	0.05	0.27*	0.15
	At 2 years lag	−0.02	0.25*	−0.25*	0.03

#### 3.5.1 Effect of Socio‐Demographic Factors on Nutrition

We did a simple$\;\chi^{2}\;$statistics analysis and the p‐value of the chi‐square statistics for children nutrition status versus each of the variables is listed in Table 2.

**Table 2:** $\chi^{2}\;$test p‐value for bivariate relation of stunting and wasting prevalence with socio‐economic and demographic variables.

Covariates	$\chi^{2}\;$ test and p‐value							
	2012 (*N* = 12774)				2019 (*N* = 13856)			
	Stunting		Wasting		Stunting		Wasting	
	$\chi^{2}$	p‐value	$\chi^{2}$	p‐value	$\chi^{2}$	p‐value	$\chi^{2}$	p‐value
Access to safe water	0.54	0.46	9.30	0.00	2.43	0.12	0.10	0.75
Access to safe toilet	115.64	0.00	0.01	0.94	70.40	0.00	2.03	0.15
Access to hand wash	17.02	0.00	0.04	0.84	114.08	0.00	8.12	0.00
Wealth index	402.10	0.00	32.15	0.00	386.31	0.00	28.86	0.00
Mother's education	331.47	0.00	12.15	0.00	266.67	0.00	7.66	0.02
Mothers age	61.12	0.00	1.94	0.164	24.38	0.00	2.29	0.13
Child's age	67.96	0.00	7.04	0.03	94.38	0.00	7.00	0.03

In both 2012 and 2019, access to safe water, safe toilets, access to handwashing facilities, wealth index, maternal education, maternal age, and child's age were significantly associated with stunting while the district level variation is apparent from the distribution of the prevalence. Wasting prevalence showed fewer significant associations, only wealth status and child's age were found as influential factors.

#### 3.5.2 Effect of Weather Variables Adjusting for Other Covariates

**Figure 4:**
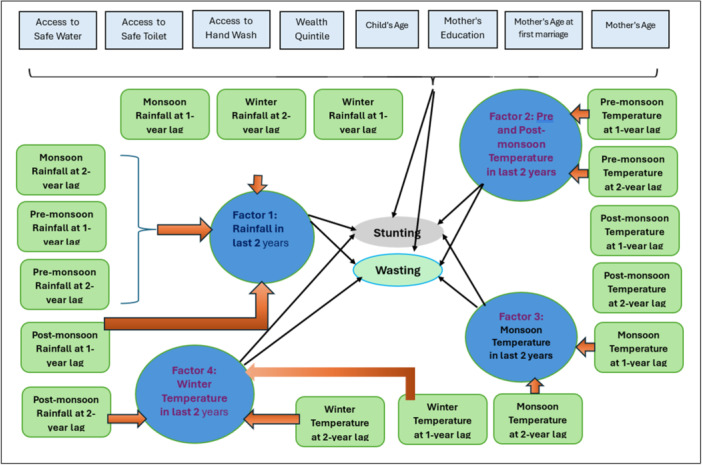
The relationship among factors and malnutrition.

Descriptive and bivariable analyses indicate that, beyond program interventions, climatic and socio‐economic factors influence malnutrition trends. The variable associations suggest that changing weather patterns may drive district‐level disparities. Understanding this context is crucial for a comprehensive assessment of how climatic variability affects child health, as illustrated in Figure 4.

Addressing these challenges requires an integrated approach and needs a clear identification of the influential weather variables factors after adjusting the effect of other factors.

The multilevel model (Table 3, full results in Annex Table 4) using FA‐derived weather factors and adjusting for individual and household characteristics showed strong fit (LR test *p*  < 0.05, Table 4). For stunting, random effects for year and district were substantial, indicating notable spatial and temporal variation (Table A5). For wasting, these effects were smaller but significant (*p* =  0.01), suggesting less variation across districts and years, reflecting more persistent patterns (Table A5).

**Table 3:** Weather factors affecting nutritional status (*N* = 9418).

Weather factors	Dependent variable			
	Stunting		Wasting	
	AOR (95% CI)	*p*‐value	AOR (95% CI)	*p*‐value
Rainfall in last 2 years	1.10 (1.03, 1.17)	0.02	1.01 (0.93, 1.10)	0.82
Pre‐ and post‐monsoon temperature in last 2 years	1.24 (1.12, 1.37)	0.00	0.95 (0.87, 1.05)	0.40
Monsoon temperature in last 2 years	0.89 (0.81, 0.97)	0.04	0.92 (0.84, 1.00)	0.09
Winter temperature in last 2 years	1.07 (0.97, 1.18)	0.28	1.02 (0.94, 1.11)	0.74

The models including weather factors showed that for stunting, the first three weather factors were statistically significant (*p* < 0.05), whereas for wasting, only the third factor was moderately significant (*p* < 0.10). As noted in Section 3.3, the three weather factors represent:

1. Factor 1: Rainfall during the previous 2 years,
2. Factor 2: Pre‐ and post‐monsoon temperature over the previous 2 years, and
3. Factor 3: Monsoon temperature over the previous 2 years.

Factor 3 was negatively associated with both stunting and wasting (OR = 0.8881), indicating that lower average monsoon temperature across the two preceding years increases the likelihood of child malnutrition. In the stunting model, Factor 2 showed a highly significant positive effect (*p* = 0.0004; OR = 1.2368), implying that higher pre‐ and post‐monsoon temperatures are linked to higher odds of stunting. Factor 1 (rainfall) was also positively significant, suggesting that increased rainfall during the preceding 2 years is associated with elevated stunting risk.

## 4 Discussion

The findings highlight the combined influence of climatic and socio‐demographic factors on child malnutrition in Bangladesh. Pre‐ and post‐monsoon temperature deviations were strongly associated with stunting, likely affecting thermoregulation, metabolism, appetite, and nutrient absorption (Desta et al. 2024). Lower‐than‐usual monsoon temperatures (Factor 3) were negatively linked to both stunting and wasting, reflecting disruptions in environmental and agricultural cycles (Shah et al. 2021).

Excessive rainfall (Factor 1) positively correlated with stunting, possibly through impacts on food security, agricultural productivity, and disease transmission (Hagos et al. 2014). These patterns reinforce that climatic anomalies—temperature and rainfall variability—amplify stunting risk directly via physiological stress and indirectly through food system disruption (Amondo et al. 2023).

Socio‐demographic determinants, informed by prior literature (Khan et al. 2023; Johnson and Brown 2014; Hasan et al. 2023; Smith et al. 2021), including child age, maternal education, economic status, geographic location, and maternal characteristics, further shape malnutrition outcomes. Examining these factors across districts and over time is critical for understanding spatial and temporal variations in stunting and wasting.

While focused on Bangladesh, the findings contribute to the global discourse linking climate variability and child nutrition, consistent with evidence from sub‐Saharan Africa, South Asia, and other low‐ and middle‐income regions. These insights can inform both national and international climate‐resilient nutrition strategies (Amondo et al. 2023; Desta et al. 2024; Khan et al. 2023).

## 5 Conclusion

This study aimed to examine how climatic variability—particularly pre‐ and post‐monsoon temperature—affects child nutrition outcomes such as stunting and wasting. The results confirmed that temperature‐related weather factors are indeed key determinants of stunting, with pre‐ and post‐monsoon temperature showing a highly significant positive effect and monsoon temperature demonstrating a negative association with both stunting and wasting. These findings directly address the first objective by establishing pre‐ and post‐monsoon temperature as critical climatic drivers of undernutrition.

By linking high‐resolution ENACTS‐BMD weather data with MICS child anthropometry using 1‐ and 2‐year seasonal lags, the analysis successfully captured the cumulative and delayed effects of climate on child nutrition. The significant associations observed for multiple weather factors underscore the importance of considering prolonged and lagged climatic influences rather than isolated short‐term variations.

The application of factor analysis and multilevel modeling effectively managed multicollinearity among correlated weather variables, enabling robust estimation of their distinct effects on stunting and wasting. This analytical framework enhanced the precision of inference and allowed for the identification of climate–nutrition relationships across districts.

Finally, the findings contribute to an improved empirical and methodological understanding of the links between climatic variability and child nutrition in Bangladesh. Although the study does not directly address policy implications, it provides a framework for future research on climate–nutrition interactions in low‐ and middle‐income settings, emphasizing the need for temporally and spatially nuanced analyses.

An integrated climate–nutrition policy is urgently needed. Strengthening climate‐resilient programs in districts prone to extreme pre‐ and post‐monsoon temperatures, along with climate‐sensitive early warning systems, can mitigate nutritional deficits. Improving water, sanitation, and maternal education remains vital. Further research on long‐term impacts and adaptive interventions will support sustainable, climate‐resilient child nutrition policies in Bangladesh.

## Potential Sources of Bias and Limitations

Climatic exposures were assigned at the district level, which may introduce measurement error due to within‐district heterogeneity and seasonal aggregation that masks short‐term extremes, potentially biasing associations toward the null. Despite adjustment for socio‐demographic covariates, residual confounding from unmeasured factors—such as diet, food security, maternal nutrition, and healthcare access—cannot be ruled out. Linking individual nutrition outcomes to district‐level climate introduces ecological limitations and may smooth localized variability. Migration could misclassify exposure, and cross‐sectional survey design limits causal inference, particularly for stunting, which reflects cumulative past conditions. Findings should be interpreted as associations.

## Author Contributions

The first author conceived the study. All authors jointly designed the research, performed data analyses, and interpreted the findings. They collaborated on drafting the manuscript and provided critical revisions. Each author has reviewed and approved the final version for submission and accepts full responsibility for the integrity and accuracy of the work, addressing any issues that may arise.

## Funding

The authors received no specific funding for this work.

## Conflicts of Interest

The authors declare no conflicts of interest.

## Policy on Using ChatGPT and Similar AI Tools

We used *ChatGPT (OpenAI, GPT‐4)* in refining the language and improving the readability of the manuscript. We declare that the tool was not used to generate, analyze, or interpret any scientific content. All the ideas, data interpretations, conclusions and recommendations are entirely the work of the authors.

## Supporting information


**Figure A1:** District‐wise Mean Temperature and aggregate Rainfall of Bangladesh during 2007‐2019. **Figure A2:** A correlation plot of all the bivariate correlations among the weather variables. **Figure A3:** Scree Plot for Dimensionality Checking. **Table A1:** Correlation among Rainfall of Different Seasons. **Table A2:** Correlation among Temperature of Different Seasons. **Table A3:** Correlation among Rainfall and Temperature of Different Seasons. **Table A4:** Results of Multi‐level Logistic Model including Weather Factors for Identifying Factors Affecting Nutritional Status (N=9418). **Table A5:** Results of Random Factor Year and District.

## Data Availability

The MICS data that support the findings of this study are openly available in UNICEF MICS Data Repository at https://mics.unicef.org/surveys. The district level weather data for the period 2007–2018 can be obtained from the Bangladesh Meteorological Department (BMD).
