# Supplementary material for: Higher Pre‐ and Post‐Monsoon Temperatures and Their Impact on Child Undernutrition in Bangladesh
Source: Matern Child Nutr. 2026 Mar 16;22(1):e70176. doi: 10.1111/mcn.70176 (PMC13344144; doi:10.1111/mcn.70176)
Supplement: Supplementary file 1 — Figure A1: District‐wise Mean Temperature and aggregate Rainfall of Bangladesh during 2007‐2019. Figure A2: A correlation plot of all the bivariate correlations among the weather variables. Figure A3: Scree Plot for Dimensionality Checking. Table A1: Correlation among Rainfall of Different Seasons. Table A2: Correlation among Temperature of Different Seasons. Table A3: Correlation among Rainfall and Temperature of Different Seasons. Table A4: Results of Multi‐level Logistic Model including Weather Factors for Identifying Factors Affecting Nutritional Status (N=9418). Table A5: Results of Random Factor Year and District. [file MCN-22-e70176-s001.docx]

Annex:

**Figure A.1: District-wise Mean Temperature and aggregate Rainfall of Bangladesh during 2007-2019**

| 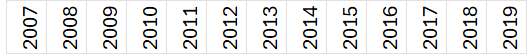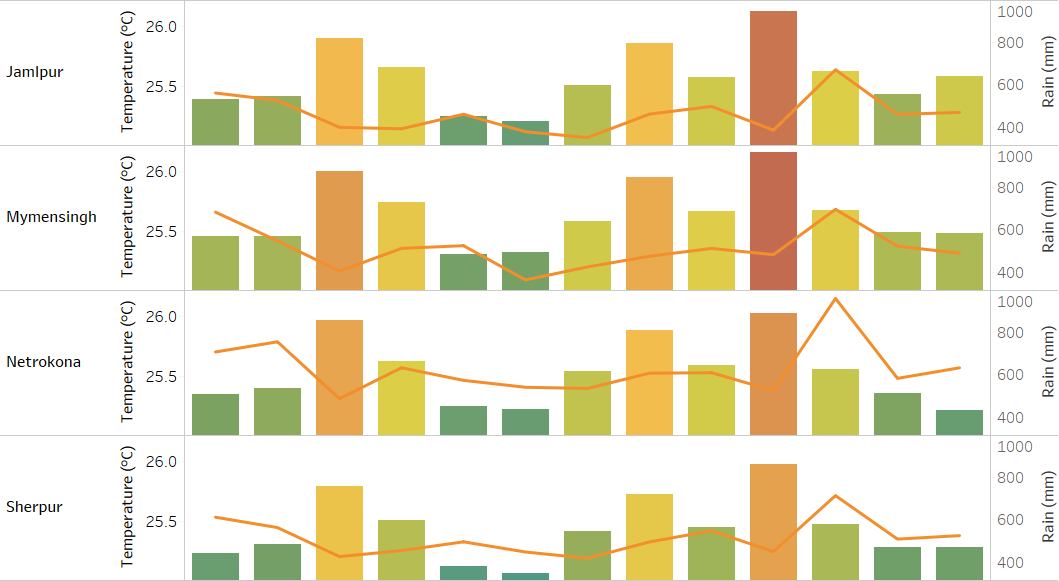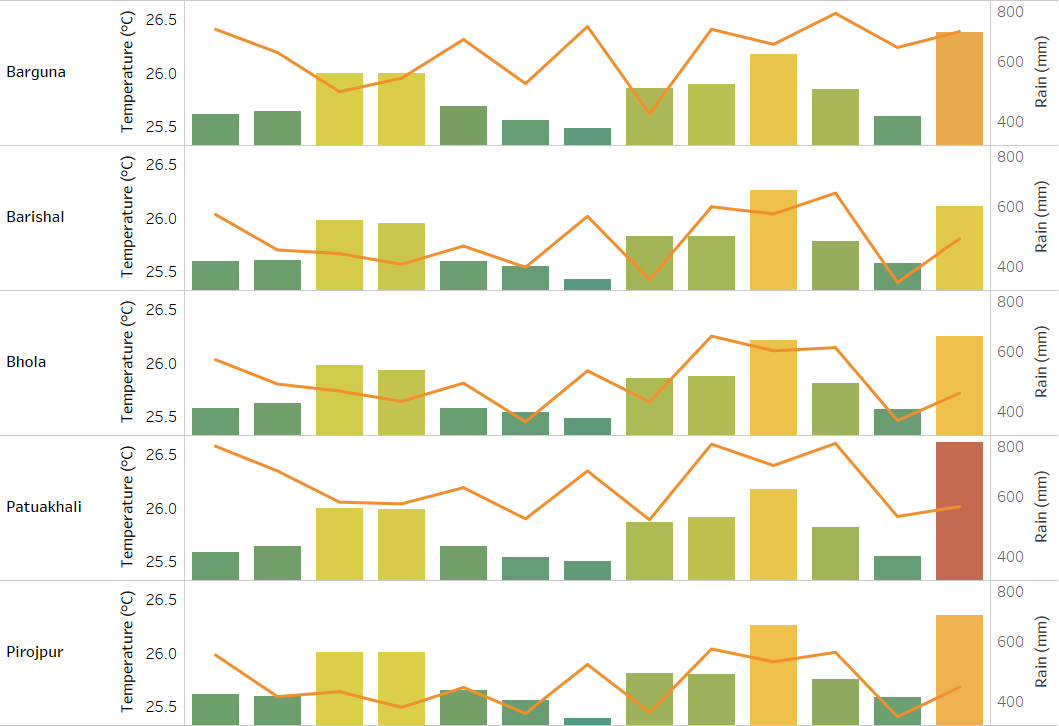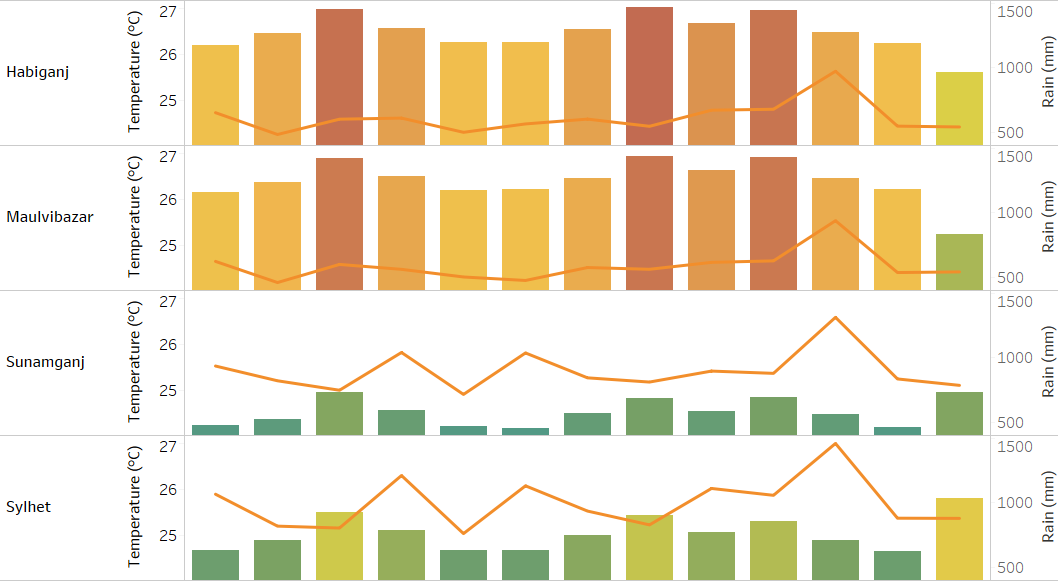 | | | 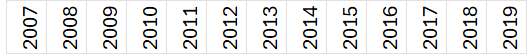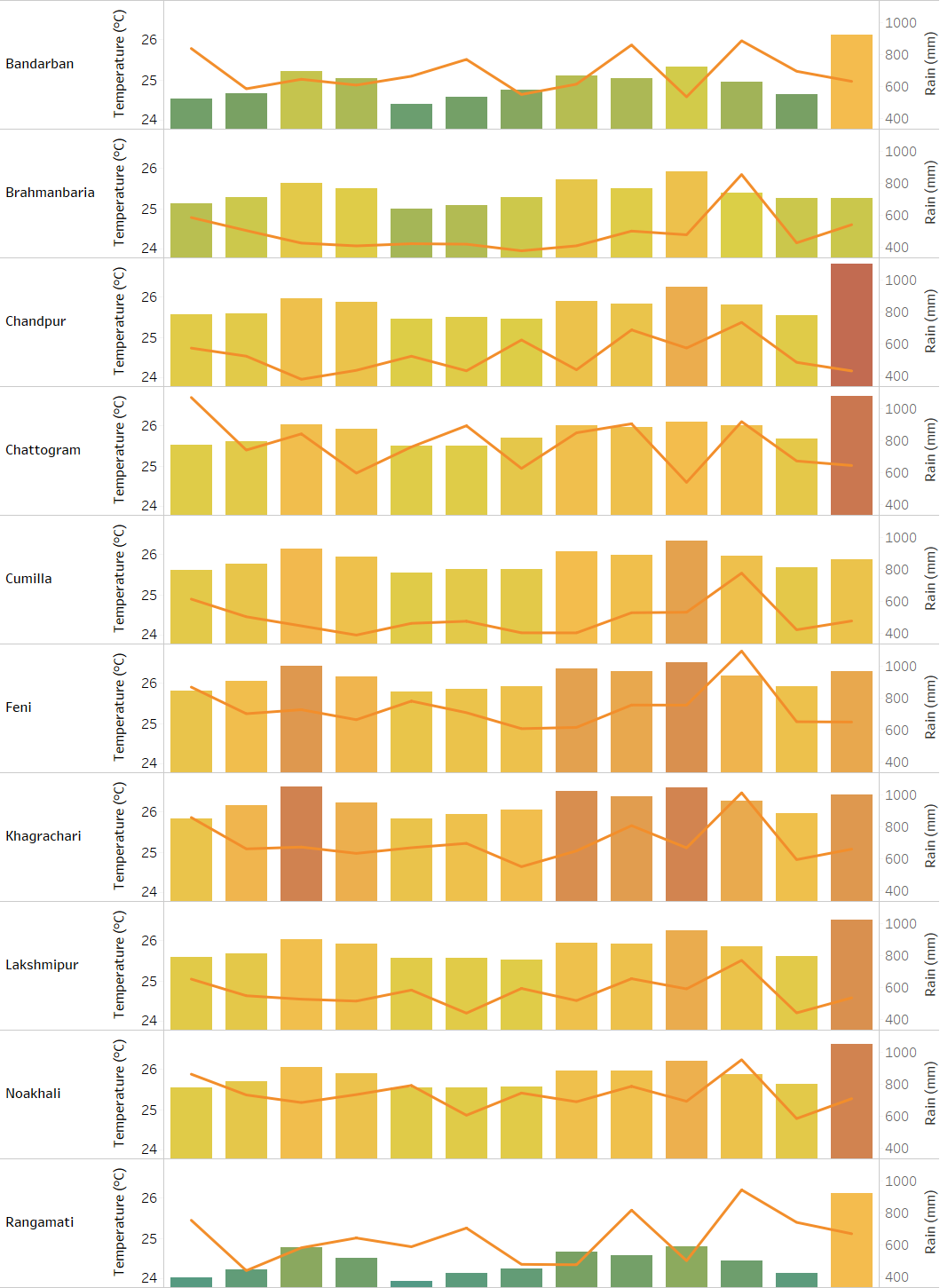 |  |
| --- | --- | --- | --- | --- |
| *The bars represent yearly mean temperature and the lines represent yearly aggregate rainfall. | | | |  |
| 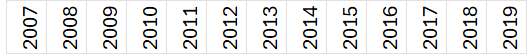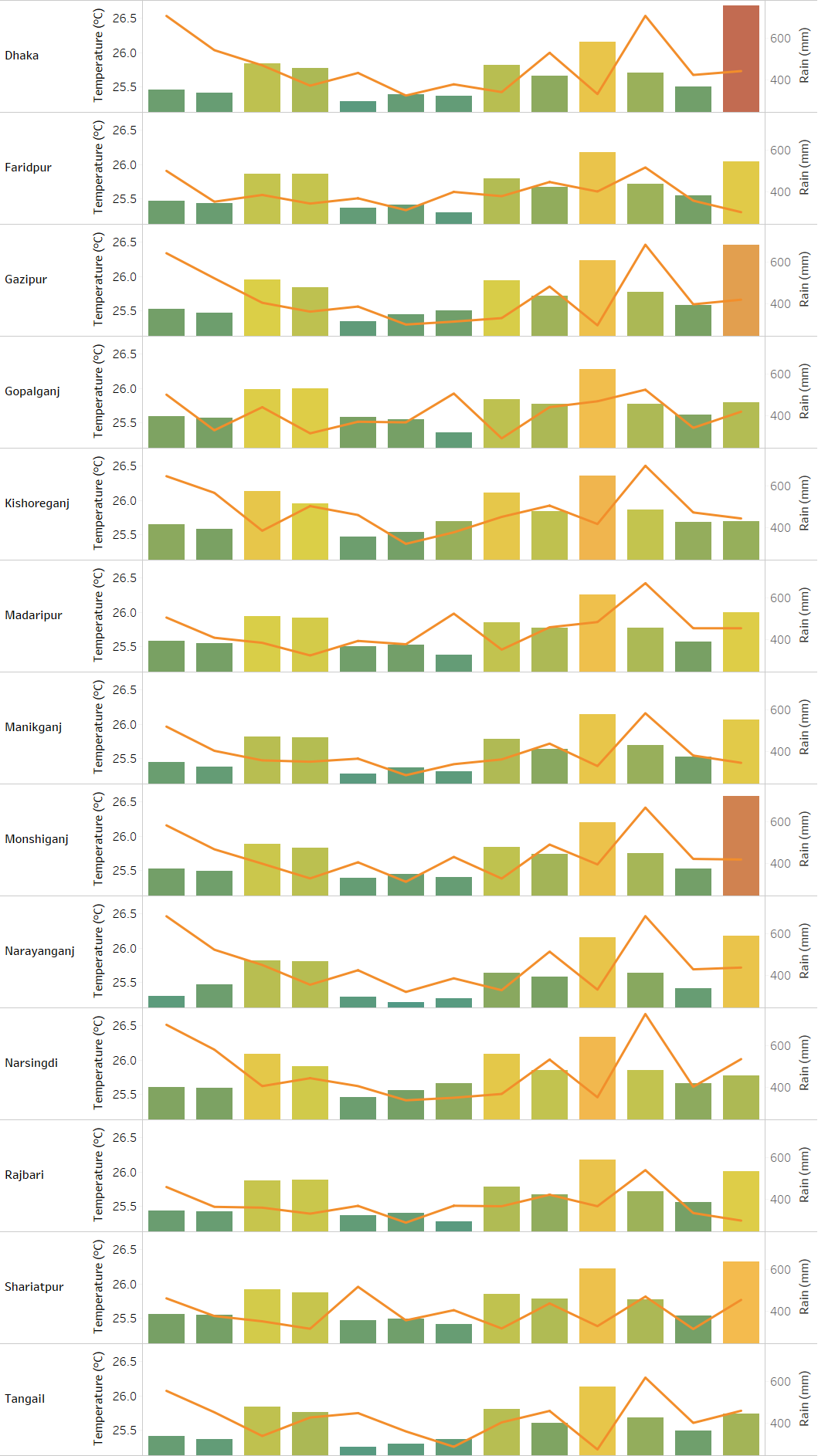 | | 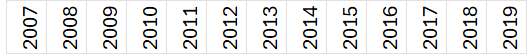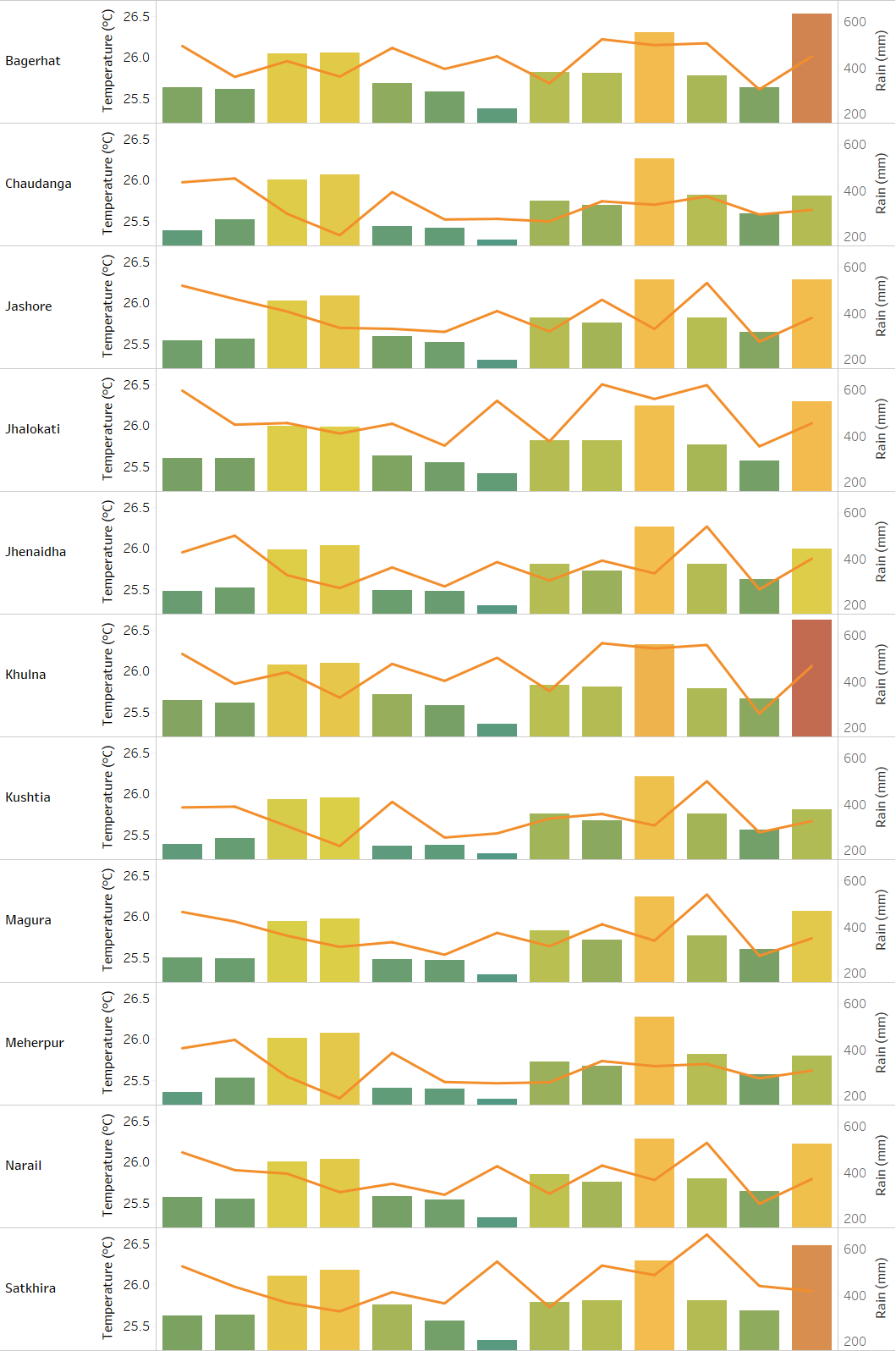 | |  |
| *The bars represent yearly mean temperature and the lines represent yearly aggregate rainfall. | | | | |
|  | |  | |  |
| 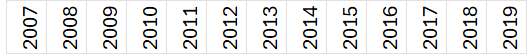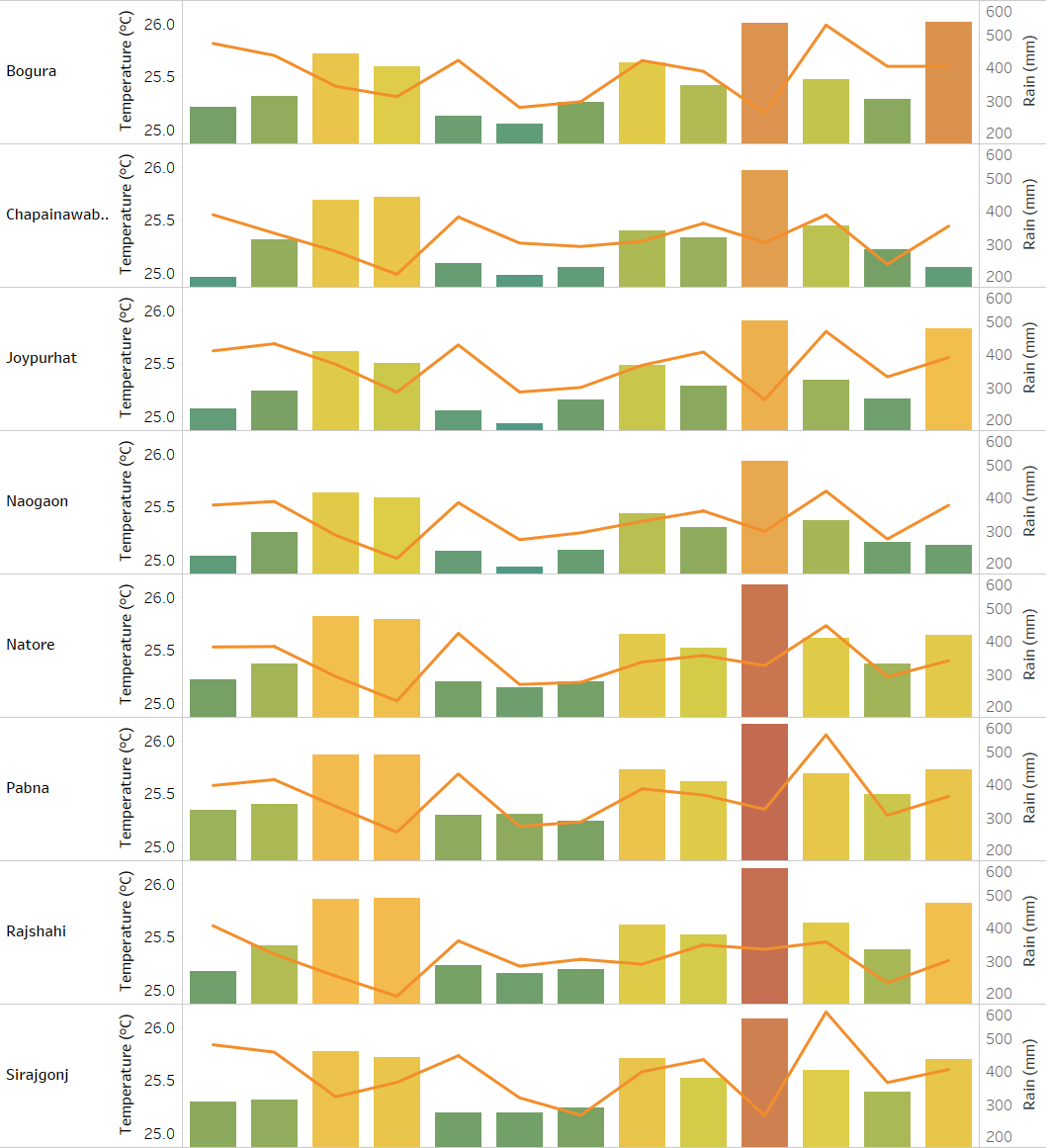 | | 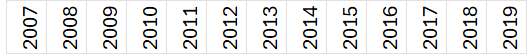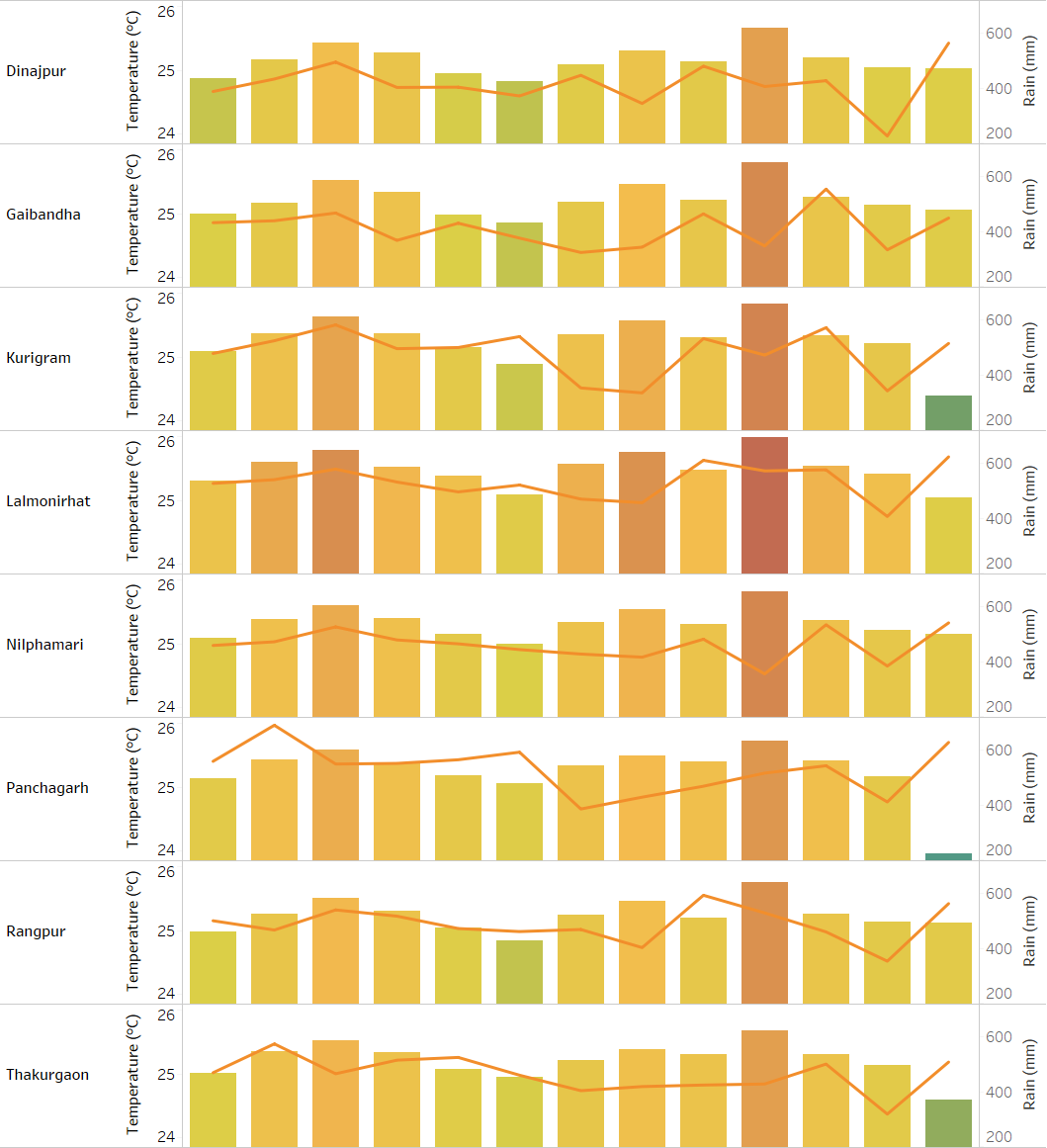 |  |  |
| *The bars represent yearly mean temperature and the lines represent yearly aggregate rainfall. | | |  |  |

**Figure A.2: A correlation plot of all the bivariate correlations among the weather variables**

| 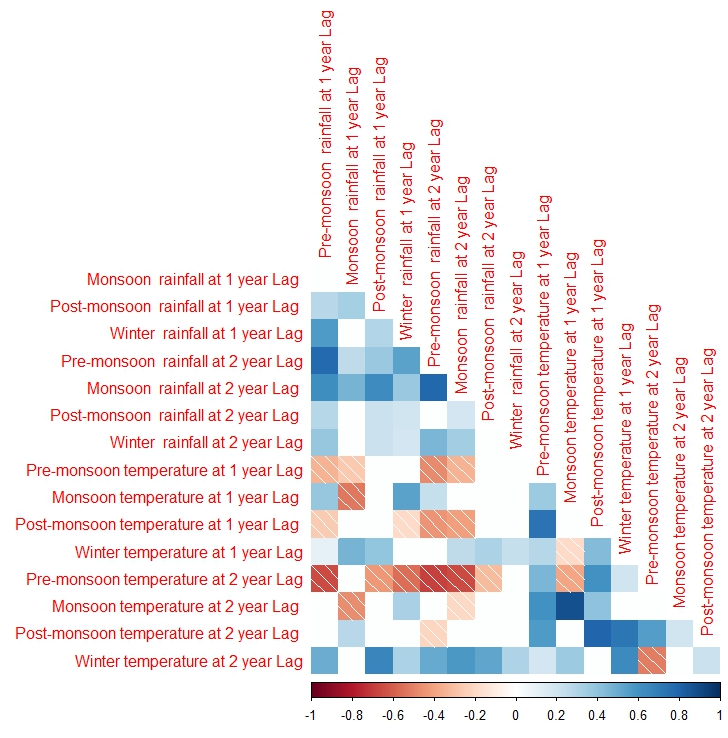 |
| --- |

**Figure A.3:** **Scree Plot for Dimensionality Checking**

| 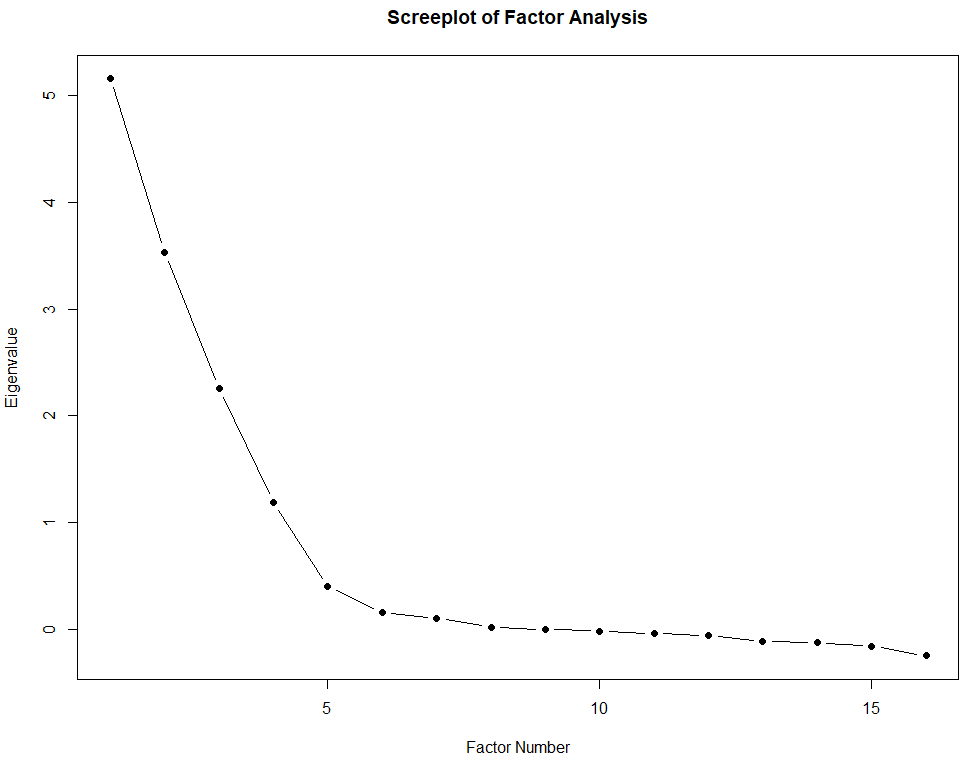 |
| --- |
|  |
|  |

\\

**Table A.1: Correlation among Rainfall of Different Seasons**

| Rainfall | Rainfall | | | | | | | |
| --- | --- | --- | --- | --- | --- | --- | --- | --- |
|  | at 1 year Lag | | | | at 2 years Lag | | | |
|  | Pre-Monsoon | Monsoon | Post-Monsoon | Winter | Pre-Monsoon | Monsoon | Post-Monsoon | Winter |
| Pre-monsoon at 1 year Lag | 1 |  |  |  |  |  |  |  |
| Monsoon at 1 year Lag | 0.01 | 1 |  |  |  |  |  |  |
| Post-monsoon at 1 year Lag | 0.28* | 0.33* | 1 |  |  |  |  |  |
| Winter at 1 year Lag | 0.57* | -0.17 | 0.30* | 1 |  |  |  |  |
| Pre-monsoon at 2 years Lag | 0.78* | 0.26* | 0.38* | 0.53* | 1 |  |  |  |
| Monsoon at 2 years Lag | 0.62* | 0.47* | 0.63* | 0.37* | 0.78* | 1 |  |  |
| Post-monsoon at 2 years Lag | 0.29* | -0.09 | 0.21* | 0.19* | 0.13 | 0.19* | 1 |  |
| Winter at 2 years Lag | 0.38* | 0.12 | 0.21* | 0.18* | 0.46* | 0.34* | 0.03 | 1 |
| ** indicates significant at 5% level of significance* | | | | | | | | |

**Table A.2: Correlation among Temperature of Different Seasons**

| Temperature | Temperature | | | | | | | |
| --- | --- | --- | --- | --- | --- | --- | --- | --- |
|  | at 1 year Lag | | | | at 2 years Lag | | | |
|  | Pre-Monsoon | Monsoon | Post-Monsoon | Winter | Pre-Monsoon | Monsoon | Post-Monsoon | Winter |
| Pre-monsoon at 1 year Lag | 1 |  |  |  |  |  |  |  |
| Monsoon at 1 year Lag | 0.37* | 1 |  |  |  |  |  |  |
| Post-monsoon at 1 year Lag | 0.73* | 0.15 | 1 |  |  |  |  |  |
| Winter at 1 year Lag | 0.28* | -0.19* | 0.43* | 1 |  |  |  |  |
| Pre-monsoon at 2 years Lag | 0.45* | -0.40* | 0.60* | 0.19* | 1 |  |  |  |
| Monsoon at 2 years Lag | 0.61* | 0.88* | 0.41* | -0.16 | 0.02 | 1 |  |  |
| Post-monsoon at 2 years Lag | 0.56* | -0.04 | 0.79* | 0.72* | 0.56* | 0.20* | 1 |  |
| Winter at 2 years Lag | 0.19* | 0.37* | 0.11 | 0.64* | -0.51* | 0.14 | 0.21* | 1 |
| ** indicates significant at 5% level of significance* | | | | | | | | |
|  | | | | | | | | |

**Table A.3: Correlation among Rainfall and Temperature of Different Seasons**

| Rainfall | Temperature | | | | | | | |
| --- | --- | --- | --- | --- | --- | --- | --- | --- |
|  | at 1 year Lag | | | | at 2 years Lag | | | |
|  | Pre-monsoon | Monsoon | Post-monsoon | Winter | Pre-monsoon | Monsoon | Post-monsoon | Winter |
| Pre-monsoon at 1 year Lag | -0.34* | 0.38* | -0.25* | 0.11 | -0.65* | 0.06 | -0.13 | 0.50* |
| Monsoon at 1 year Lag | -0.27* | -0.52* | -0.16 | 0.47* | 0.14 | -0.47* | 0.28* | 0.05 |
| Post-monsoon at 1 year Lag | -0.00 | 0.07 | -0.17 | 0.39* | -0.44* | -0.09 | 0.06 | 0.65* |
| Winter at 1 year Lag | -0.14 | 0.53* | -0.19* | -0.15 | -0.55* | 0.33* | -0.17 | 0.32* |
| Monsoon at 2 years Lag | -0.47* | 0.23* | -0.45* | 0.15 | -0.68* | -0.10 | -0.21* | 0.50* |
| Pre-monsoon at 2 years Lag | -0.34* | 0.11 | -0.42* | 0.26* | -0.66* | -0.21* | -0.16 | 0.58* |
| Post-monsoon at 2 years Lag | 0.14 | 0.12 | 0.16 | 0.31* | -0.31* | 0.01 | 0.07 | 0.52* |
| Winter at 2 years Lag | -0.17 | -0.02 | -0.01 | 0.23* | -0.12 | -0.11 | 0.06 | 0.31* |
| ** indicates significant at 5% level of significance* | | | | | | | | |

**Table A4: Results of Multi-level Logistic Model including Weather Factors for Identifying Factors Affecting Nutritional Status (N=9418)**

| Predictors | Dependent Variable | | | | | |
| --- | --- | --- | --- | --- | --- | --- |
|  | Stunting | | | Wasting | | |
|  | OR | p-value | CI for OR | OR | p-value | CI for OR |
| **Weather Characteristics** |  |  |  |  |  |  |
| Weather Factor 1 (Rainfall in last 2 years) | 1.10 | 0.02 | (1.03, 1.17) | 1.01 | 0.82 | (0.93, 1.10) |
| Weather Factor 2 (Pre and post monsoon temperature in last 2 years) | 1.24 | 0.00 | (1.12, 1.37) | 0.95 | 0.40 | (0.87, 1.05) |
| Weather Factor 3 (Monsoon temperature in last 2 years) | 0.89 | 0.04 | (0.81, 0.97) | 0.92 | 0.09 | (0.84, 1.00) |
| Weather Factor 4 (Winter temperature in last 2 years) | 1.07 | 0.28 | (0.97, 1.18) | 1.02 | 0.74 | (0.94, 1.11) |
| **Individual Characteristics** |  |  |  |  |  |  |
| Child's Age (ref: 24-35): 36-47 | 0.89 | 0.03 | (0.81, 0.97) | 1.04 | 0.65 | (0.90, 1.21) |
| Child's Age (ref: 24-35): 48-59 | 0.67 | 0 | (0.61, 0.74) | 1.05 | 0.60 | (0.90, 1.22) |
| **Household Characteristics** |  |  |  |  |  |  |
| Access to Safe Water | 0.89 | 0.24 | (0.75, 1.05) | 0.79 | 0.11 | (0.62, 1.01) |
| Access to Safe Toilet | 0.85 | 0.02 | (0.77, 0.95) | 1.11 | 0.34 | (0.93, 1.32) |
| Access to Hand Wash | 0.98 | 0.77 | (0.87, 1.10) | 0.80 | 0.04 | (0.68, 0.96) |
| Wealth Quintile (ref: poorest): poor | 0.87 | 0.04 | (0.77, 0.97) | 1.08 | 0.48 | (0.90, 1.31) |
| Wealth Quintile (ref: poorest): mid | 0.61 | 0 | (0.54, 0.69) | 1.02 | 0.86 | (0.84, 1.25) |
| Wealth Quintile (ref: poorest): rich | 0.54 | 0 | (0.47, 0.62) | 0.93 | 0.56 | (0.75, 1.15) |
| Wealth Quintile (ref: poorest): richest | 0.33 | 0 | (0.29, 0.39) | 0.84 | 0.22 | (0.66, 1.06) |
| **Maternal Characteristics** |  |  |  |  |  |  |
| Mother's Education (ref: illiterate): Primary | 0.83 | 0.01 | (0.74, 0.92) | 1.22 | 0.07 | (1.02, 1.47) |
| Mother's Education (ref: illiterate): Secondary and above | 0.65 | 0 | (0.56, 0.74) | 1.04 | 0.78 | (0.84, 1.28) |
| Mother's Age At First Marriage | 1.00 | 0.74 | (0.99, 1.02) | 0.99 | 0.52 | (0.97, 1.01) |
| Mother's Age (ref: 15-19): 20-24 | 0.81 | 0.13 | (0.65, 1.02) | 0.83 | 0.39 | (0.58, 1.18) |
| Mother's Age (ref: 15-19): 25-29 | 0.72 | 0.02 | (0.58, 0.90) | 0.97 | 0.90 | (0.68, 1.39) |
| Mother's Age (ref: 15-19): 30-34 | 0.76 | 0.05 | (0.60, 0.96) | 1.09 | 0.71 | (0.76, 1.56) |
| Mother's Age (ref: 15-19): 35-39 | 0.69 | 0.01 | (0.53, 0.88) | 0.81 | 0.39 | (0.55, 1.21) |
| Mother's Age (ref: 15-19): 40-44 | 0.62 | 0.01 | (0.45, 0.84) | 0.99 | 0.99 | (0.62, 1.59) |
| Mother's Age (ref: 15-19): 45-49 | 0.71 | 0.14 | (0.48, 1.04) | 1.08 | 0.83 | (0.60, 1.95) |
| **Constant** | 2.22 | 0.05 | (1.13, 4.37) | 0.14 | 0 | (0.09, 0.24) |

**Table A5: Results of Random Factor Year and District**

|  | Stunting | Wasting |
| --- | --- | --- |
| Estimated Variance Components of year ($\sigma_{\mathrm{Year}}^{2}$) | 0.25 | 0.00 |
| Estimated Variance Components of District ($\sigma_{\mathrm{District}}^{2}$) | 0.05 | 0.00 |
| LR test vs. logistic model | $\chi^{2}$ = 846.78; Sig. = 0.00 | $\chi^{2}$ = 40.08; Sig. = 0.01 |
